# Low‐Dose Cone‐Beam Computed Tomography in Swedish Pediatric Patients With Alveolar Clefts Following Alveolar Bone Grafting—A Clinical Study

**DOI:** 10.1002/cre2.70021

**Published:** 2024-11-04

**Authors:** António Vicente, Josefine Cederhag, Nilofar Rashidi, Anna‐Paulina Wiedel, Magnus Becker, Susanne Brogårdh‐Roth, Xie‐Qi Shi, Kristina Hellén‐Halme

**Affiliations:** ^1^ Department of Oral and Maxillofacial Radiology, Faculty of Odontology Malmö University Malmö Sweden; ^2^ Department of Oral and Maxillofacial Surgery Skåne University Hospital Malmö Sweden; ^3^ Department of Orthodontics, Faculty of Odontology Malmö University Malmö Sweden; ^4^ Department of Clinical Sciences in Malmö Lund University Malmö Sweden; ^5^ Department of Plastic and Reconstructive Surgery Skåne University Hospital Malmö Sweden; ^6^ Department of Paediatric Dentistry, Faculty of Odontology Malmö University Malmö Sweden; ^7^ Section of Oral and Maxillofacial Radiology, Department of Clinical Dentistry University of Bergen Bergen Norway

**Keywords:** alveolar bone grafting, cone‐beam computed tomography, orofacial cleft, radiation

## Abstract

**Objectives:**

The aim of this study was to investigate whether a low‐dose cone‐beam computed tomography (CBCT) protocol provides diagnostically acceptable image quality for assessing bone healing after alveolar bone grafting.

**Material and Methods:**

The study cohort comprised 11 patients (aged 7–14 years) with orofacial clefts who had undergone alveolar bone grafting at Skåne University Hospital in Malmö, Sweden. During the postsurgical follow‐up at 6 months, each patient was assessed twice: once with a standard‐dose CBCT protocol and once with a low‐dose CBCT protocol, which in total corresponds to one CBCT examination made with the exposure settings recommended by the manufacturer. Among others, the assessed parameters included subjective image quality, as well as bone graft height, thickness, and integration.

**Results:**

No significant differences were found between the standard‐ and low‐dose protocols for most parameters (*p* > 0.05). Exceptions included subjective image quality (one observer, *p* = 0.05) and confidence levels during the assessment (three observers, *p* = 0.01, 0.01, 0.02).

**Conclusions:**

The low‐dose protocol yielded adequate image quality for postoperative CBCT healing assessment in patients who have undergone alveolar bone grafting. However, the confidence level of observers during the assessment with the low‐dose protocol was reduced. This study is registered on ClinicalTrials.gov (NCT06395077).

**Clinical Trial Registration:**

This study is registered on ClinicalTrials.gov (NCT06395077).

## 1 Introduction

Orofacial clefts are among the most common birth defects, (Salari et al. 2022), and their etiology is multifactorial (Shkoukani, Chen, and Vong 2013). An orofacial cleft results from developmental disturbances of the maxillae and palate during the first 3 months of gestation (Shkoukani, Chen, and Vong 2013). Alveolar bone grafting (ABG), a surgical procedure that uses autogenous bone grafts to close the oronasal fistulae, is usually done when the child is between 9 and 12 years of age (Abramson et al. 2015). The systematic review by De Mulder et al. concluded that the canine development stage outweighs biological age in importance for determining the optimal timing for ABG (De Mulder et al. 2019).

One of the primary goals of ABG is to build a bone bridge that will facilitate tooth eruption and subsequent orthodontic tooth movement (De Mulder et al. 2019). After surgery, most patients undergo a radiographic examination to assess the surgical outcome in aspects such as the bone fill of the cleft and the eruption status of the lateral incisor or canine adjacent to the cleft (Oberoi et al. 2009). Traditionally, this evaluation was made with two‐dimensional (2D) imaging, which only permits bone height measurement and does not allow estimation of bone thickness in the buccopalatal dimension (Oberoi et al. 2009). Furthermore, overestimations (De Mulder et al. 2019; Wiedel et al. 2021) and underestimations (Wiedel et al. 2021) of the bone graft outcome often occur in 2D post‐graft evaluation due to distortion, overlap of anatomical structures, and inability to measure the buccopalatal dimension (Ko et al. 2024; Kamperos et al. 2020; Garib et al. 2017). The assessment of bone thickness in the buccopalatal direction is important for treatment planning and follow‐up, as resorption occurs not only vertically but also in the horizontal plane (De Mulder et al. 2019).

Following the introduction of three‐dimensional imaging in dentistry, there has been a change in radiographic methods for assessing ABG, with cone‐beam computed tomography (CBCT) gaining popularity (De Mulder et al. 2019). Indeed, 3D evaluations seem more precise and trustworthy (Ko et al. 2024). SedentexCT guidelines (European Commission 2012) state that CBCT is a justifiable radiographic method in patients with orofacial clefts, as the effective dose to the patients is significantly lower compared to medical CT. Thus, when needed, CBCT is a valuable clinical method for assessing the success of ABG, including bone graft height, buccopalatal thickness of the bone graft, and the difference between sides in nasal floor height (Wiedel et al. 2021; Kamperos et al. 2020; Suomalainen et al. 2014).

The higher radiation dose from CBCT compared to traditional 2D examinations warrants careful consideration, particularly in children. Children are more vulnerable to ionizing radiation than adults due to their developing tissues (Van Acker et al. 2020). Jacobs et al. estimated that orofacial cleft patients may have a higher lifetime radiation exposure from dental radiographs compared to non‐orofacial cleft patients (Jacobs et al. 2018). The recent ALADAIP principle (As Low As Diagnostically Acceptable being Indication‐oriented and Patient‐specific), which has its roots in the ALADA principle (As Low As Diagnostically Acceptable), addresses pediatric patients in particular and advocates that exposure parameters be selected according to justification and the characteristics of the patient in CBCT imaging. When this principle is followed, diagnostically acceptable images are produced with the lowest possible radiation dose (Hung et al. 2022; Oenning et al. 2018).

CBCT studies in other clinical contexts, such as the Iskanderani et al. study on temporomandibular joint diagnoses (Iskanderani et al. 2020) and the Cederhag et al. study on pre‐surgical assessment of mandibular third molars (Cederhag et al. 2023), have reported acceptable image quality using lower radiation doses than those used in clinical standard protocols. Yeung et al. concluded that a low‐dose protocol for CBCT should be considered in orofacial cleft evaluations (Yeung, Jacobs, and Bornstein 2019). Additionally, Lemberger et al. showed in 2024 that an ultra‐low‐dose protocol provides sufficient image quality for pre‐ and postoperative assessment of ABG in patients with orofacial clefts (Lemberger et al. 2024).

Guidelines on the use of low‐dose CBCT protocols in dental practice (Yeung, Jacobs, and Bornstein 2019; Charuakkra et al. 2023) and clinical studies employing them (Yeung, Jacobs, and Bornstein 2019) are sparse, in particular concerning children (Yeung, Jacobs, and Bornstein 2019; Pauwels et al. 2017). Thus, the present study was designed to determine whether a low‐dose CBCT protocol could provide diagnostically acceptable images for assessing bone healing after ABG. Our hypothesis was that CBCT image quality is adequate for monitoring post‐ABG healing when a low‐dose protocol is used. The use of an optimized CBCT protocol, with the attendant radiation dose reduction, has clear positive consequences for patients with orofacial clefts and society.

## 2 Materials and Methods

### 2.1 Ethical Considerations

The Swedish Ethical Review Authority approved the present study (daybook no. [Dnr.] 2020‐06790), which followed the principles of the Declaration of Helsinki throughout. Our study design also followed the International Commission on Radiological Protection (ICRP) guidelines (International Commission on Radiological Protect 1992) for research projects. All participants and their guardians signed an informed consent form after receiving information about the protocol and the benefits of the study. The study is registered on ClinicalTrials.gov (NCT06395077).

### 2.2 Study Design and Participants

All patients with orofacial clefts who had undergone ABG at Skåne University Hospital in Malmö, Sweden, and who had been referred for a radiographic examination control after at least 6 months in 2021 and in 2022, were invited in sequence during that period to participate in the study. Eleven patients (aged 7–14 years, four girls; unilateral clefts: 8; bilateral clefts: 3) accepted the invitation. Bilateral clefts were treated as two separate cleft cases in this study; thus, we analyzed 14 cleft cases. The CBCT exposure settings recommended by the manufacturer (Accuitomo 170; J Morita Corp., Kyoto, Japan) for these cases are usually 90 kV, 7 mA, and full rotation scan mode (360°, 17.5 s). In the present study, two CBCT examinations were conducted for each patient during the same appointment: one examination followed the standard‐dose protocol (exposure settings: 90 kV, 5 mA, half rotation scan mode [180°, 9.4 s]), and one followed the low‐dose protocol (90 kV, 2 mA, half rotation scan mode [180°, 9.4 s]). In total, the estimated dose did not exceed that of a single scan with the manufacturer's recommended exposure settings. The field of view (FOV) was 6.0 cm × 6.0 cm, the rotation mode was 180°, and the voxel size was 0.125 mm. Volume was reconstructed in slices of 1‐mm thickness and interval. The dose area products (DAPs) were 423 mGy cm^2^ for the standard‐dose protocol and 172 mGy cm^2^ for the low‐dose protocol.

### 2.3 CBCT Evaluations

**Figure 1:**
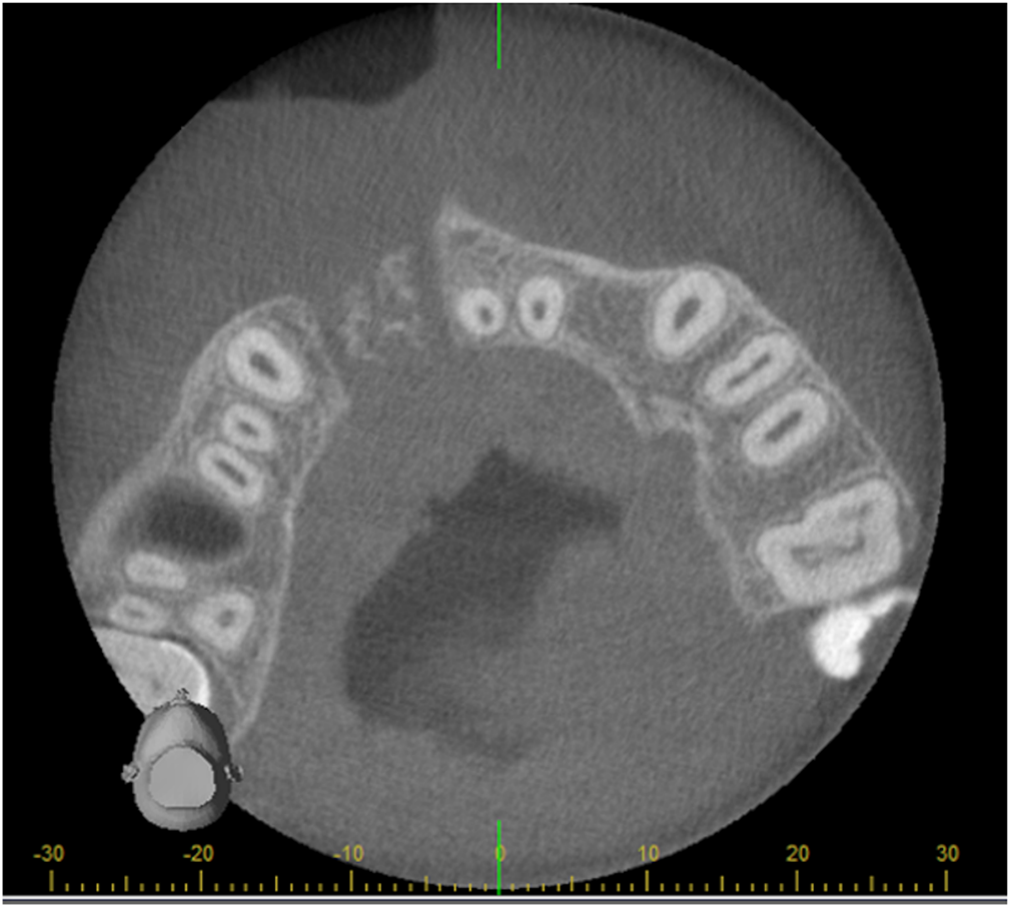
Bone integration following alveolar bone grafting. This example depicts a bone island, indicating a lack of bone integration.

**Figure 2:**
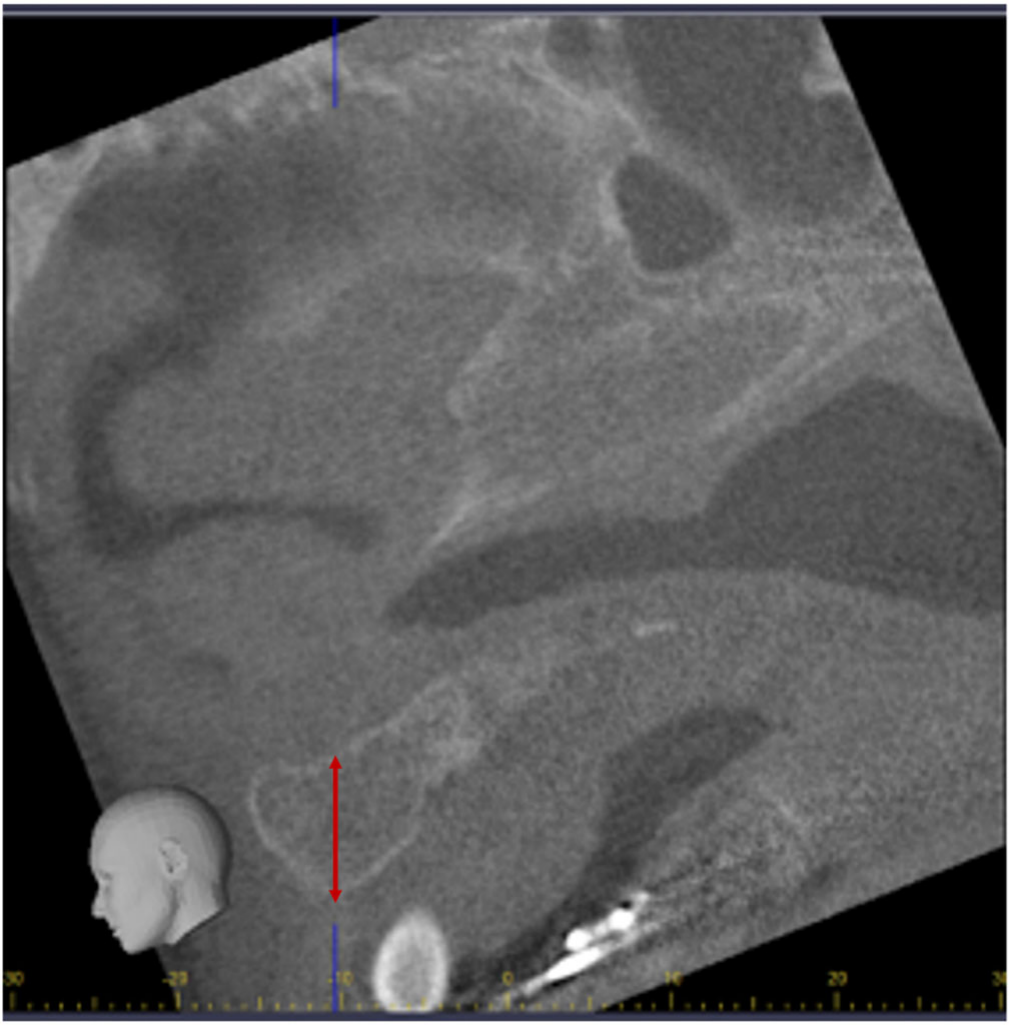
Six‐month postoperative graft height following alveolar bone grafting.

**Figure 3:**
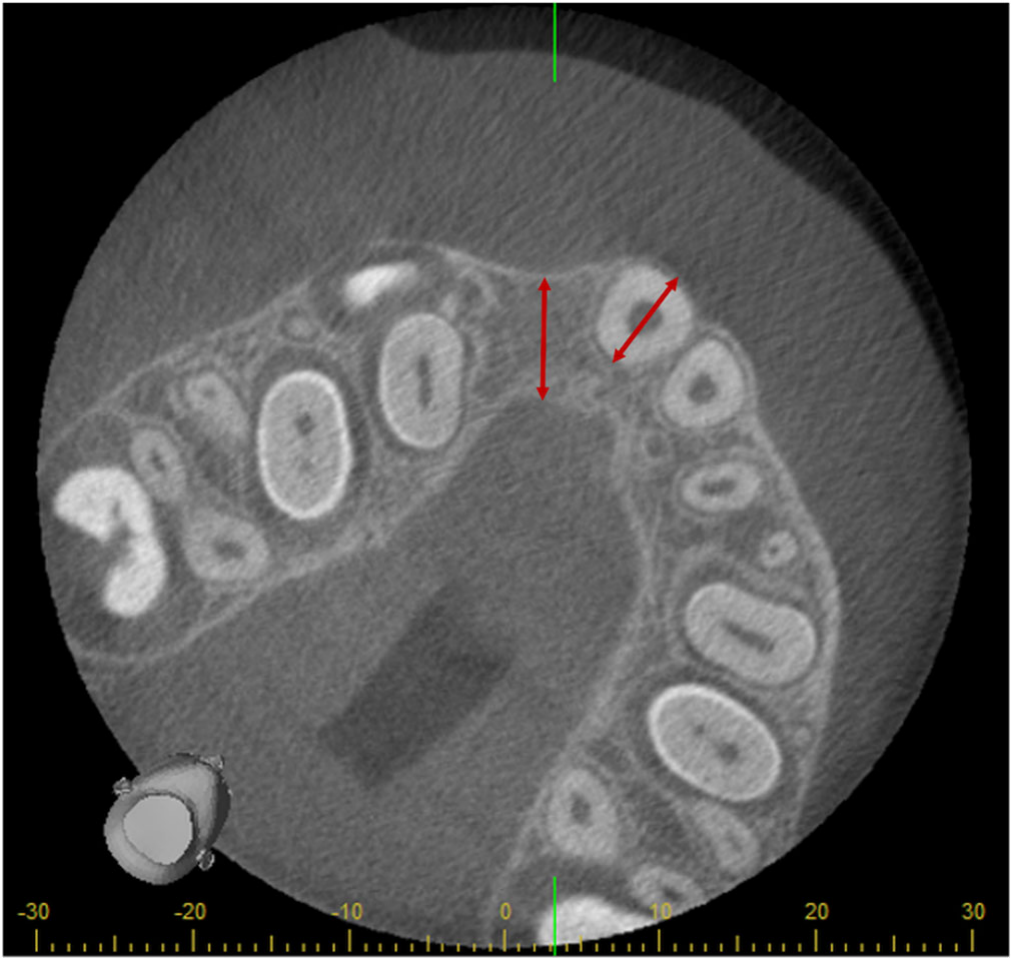
Comparison of graft thickness with the root width of the tooth adjacent to the cleft following alveolar bone grafting.

**Figure 4:**
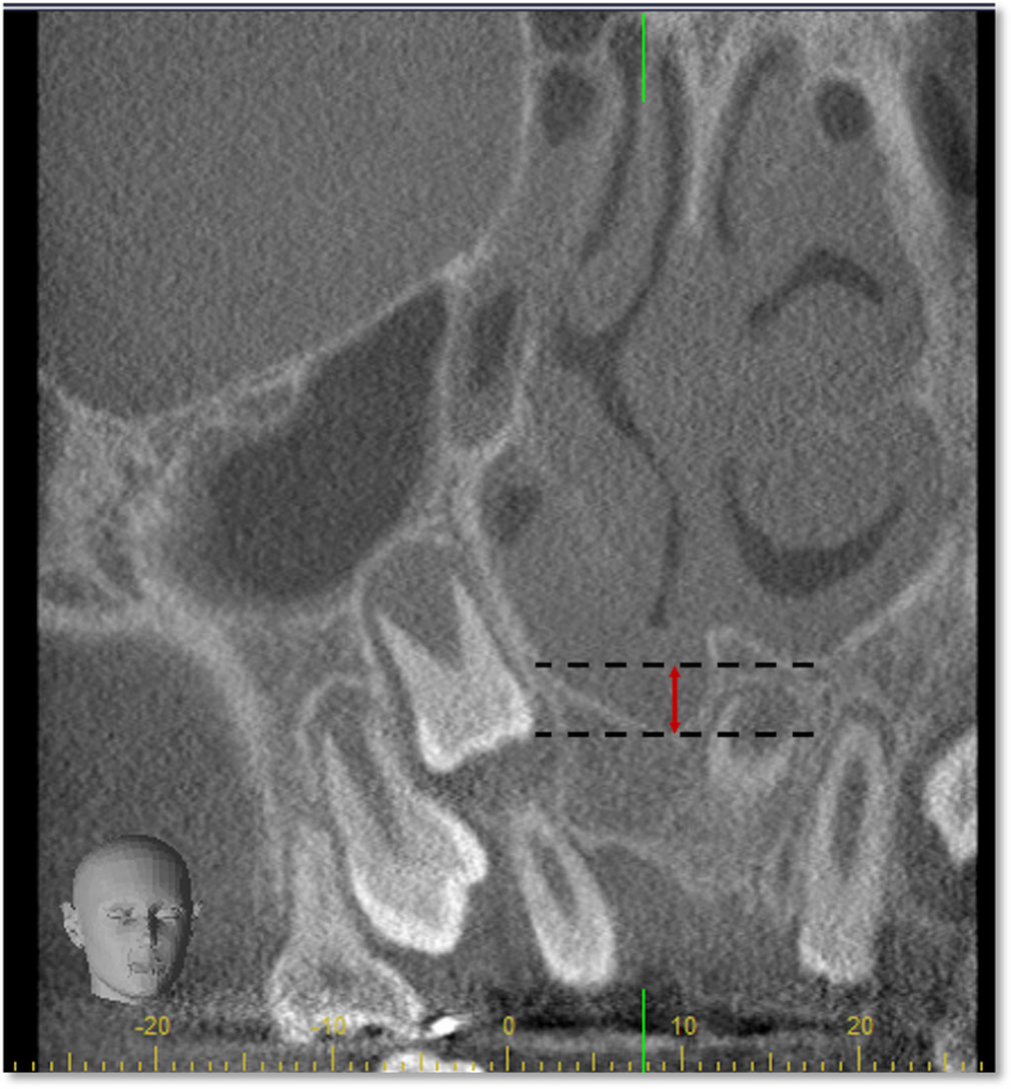
Difference in 6‐month postoperative nasal floor height between sides following alveolar bone grafting.

CBCT examinations were exported from the hospital's image database and randomly coded before the evaluation, ensuring that the observers were blinded to the examination protocol. Four calibrated oral and maxillofacial radiologists assessed the CBCT examinations: two had over 10 years of experience and two had 5 years of experience in CBCT evaluation. Evaluations were made in dimmed ambient light (< 50 lux) using the i‐Dixel One Volume Viewer imaging software (3DX Integrated Information System, 3DXD version 2.5.4.10213; J Morita Corp., Kyoto, Japan) on a calibrated monitor (Barco, 6MP, Kortrijk, Belgium). The observers had access to all of the software tools so that they could adjust as desired. All measurements were performed using the i‐Dixel One Volume Viewer imaging software. To calculate intra‐observer agreement, all observers re‐evaluated all cases at an interval of 1 month or more. Table 1 summarizes the assessed parameters. Figures 1, 2, 3, 4 illustrate examples.

**Table 1:** Assessed parameters with grading options in the cone‐beam computed tomography examinations.

Parameter	Grading options
Graft integration	Well integrated
	Partly integrated (mesial or distal/superior or inferior wall of the cleft is filled with bone)
	Not integrated (bone island)
Graft height (measured in the center of the graft)	(millimeters)
Graft thickness in the buccopalatal dimension (measured in the middle third and periapical third of the root of the tooth adjacent to the cleft)	The width of the bone bridge is as wide as, or wider than, the width of the root of the tooth adjacent to the cleft
	The width of the bone bridge is less than the width of the root of the tooth adjacent to the cleft
	No detectable bone is present
Postoperative nasal floor height difference between sides (unilateral clefts only)	(millimeters)
Subjective image quality	Diagnostically acceptable
	Diagnostically doubtful
	Diagnostically unacceptable
Confidence level during the evaluation	Very confident
	Confident
	Doubtful

### 2.4 Statistical Analysis

A power analysis indicated that 13 were the minimum number of cases required to achieve a power of 0.8 and a significance level of *p* ≤ 0.05.

All data were analyzed using the Statistical Package for the Social Sciences (IBM SPSS Statistics, version 29.0; IBM Corp, Armonk, NY).

Pairwise comparisons of the standard‐ and low‐dose protocols were made for each parameter and observer. The Wilcoxon signed‐rank test was used to assess significant differences between the two protocols for all parameters except graft height and postoperative nasal floor differences in height between sides; the *t*‐test was used for these. Cohen's weighted kappa was used to evaluate intra‐ and inter‐observer agreement for all parameters, except graft height and postoperative nasal floor differences in height between sides; for these two, the intraclass correlation coefficient (ICC) was used. The Landis and Koch scale (Landis and Koch 1977) was used to interpret Cohen's weighted kappa, and the ICC was evaluated according to Koo and Li (2016). Significance was set at *p* ≤ 0.05.

## 3 Results

**Figure 5:**
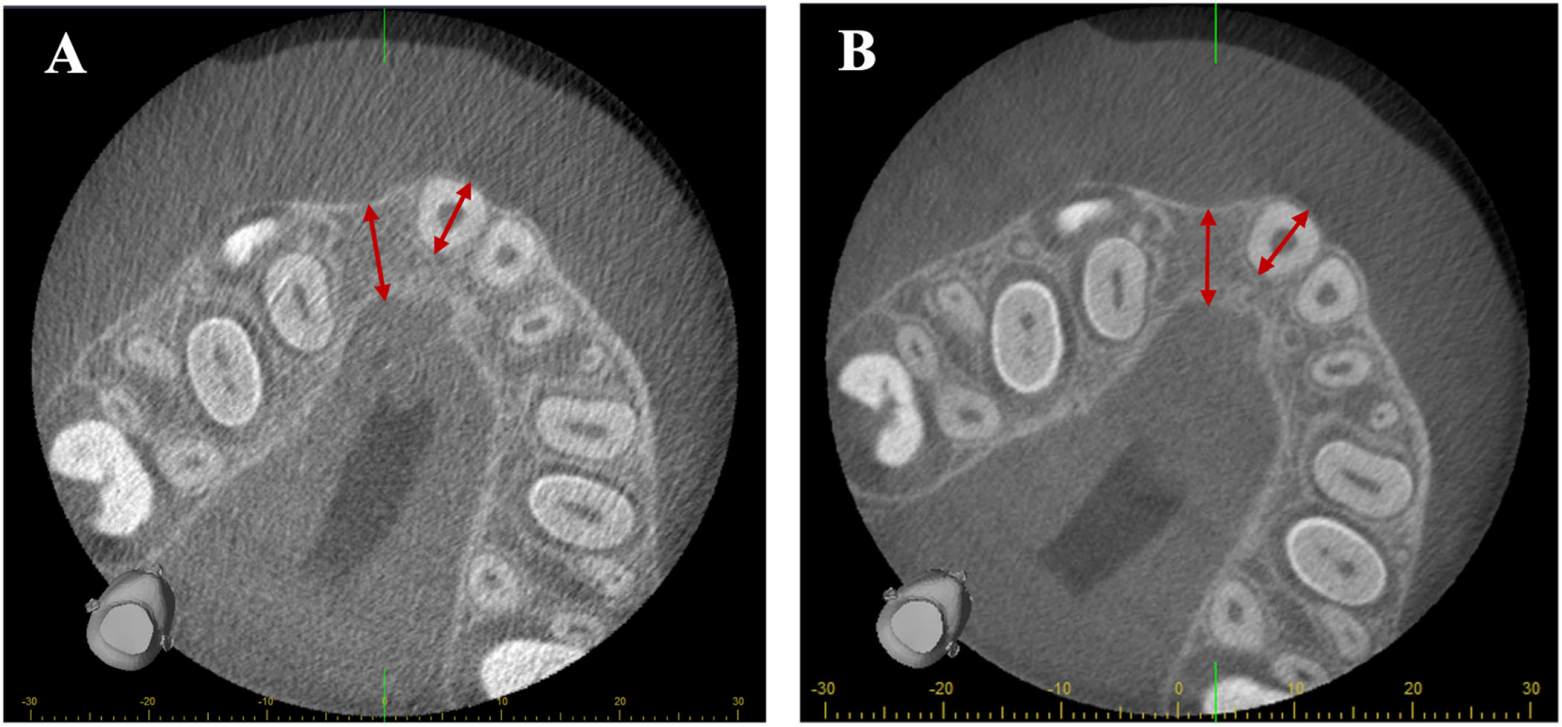
CBCT examinations showing the comparison of graft thickness with the root width of the tooth adjacent to the cleft following alveolar bone grafting made with (A) a low‐dose (2 mA) protocol and (B) a standard‐dose protocol (5 mA).

No significant differences were found between the standard‐ and low‐dose protocols for most assessed parameters, for any of the observers (Table 2). Figure 5 shows CBCT examinations of the same patient made with the two different protocols. However, significant differences occurred for subjective image quality (one observer) and in confidence level for image interpretation (three observers; Table 2).

**Table 2:** Pairwise comparisons of the standard‐ and low‐dose protocols among the four observers for the parameters assessed at least 6 months, or more, following alveolar bone grafting.

Assessed parameters	Observer			
	(*p*‐value)			
	1	2	3	4
Graft integration^a^	0.32	1.00	0.32	1.00
Graft height^b^	0.18	0.30	0.95	0.15
Graft thickness—middle^a^	0.18	0.66	0.66	0.32
Graft thickness—periapical^a^	0.32	1.00	1.00	0.32
Postoperative nasal floor height difference between sides^b^	0.61	0.18	0.17	0.67
Subjective image quality^a^	0.16	0.32	0.08	0.05*
Confidence level during the evaluation^a^	0.01*	0.32	0.01*	0.02*

^a^ Wilcoxon signed‐rank test.

^b^ *t*‐test.

* Statistically significant difference (*p* ≤ 0.05).

No observer considered any image in either protocol to be diagnostically unacceptable. In between two and five of the 14 cases, the observers found subjective image quality to be diagnostically doubtful for the low‐dose protocol compared to one for the standard‐dose protocol. Self‐ratings of confidence level during the evaluation were considered doubtful in between one to three cases for the low‐dose protocol images and in one for the standard‐dose protocol images.

Table 3 presents the kappas for inter‐observer agreement and ICCs. The two oral and maxillofacial radiologists with more experience in CBCT evaluation presented higher inter‐observer agreement for the low‐dose protocol.

**Table 3:** Inter‐observer agreement.

Assessed parameters	Range (min–max)		Mean		Results interpretation based on four observers	
	SD	LD	SD	LD	SD	LD
Graft integration^a^	0.95–1.00	1.00–1.00	0.97	1.00	Almost perfect	Almost perfect
Graft height^b^	0.75–0.95	0.69–0.86^c^	0.88	0.77	Good	Good
Graft thickness—middle^a^	0.21–0.88^c^	−0.04 to 0.77^c^	0.54	0.23	Moderate	Fair
Graft thickness—periapical^a^	0.04–0.61^c^	0.61–1.00	0.36	0.74	Fair	Substantial
Post‐operative nasal floor height difference between sides^b^	0.95–0.99	0.97–0.99	0.96	0.98	Excellent	Excellent
Subjective image quality^a^	1.00–1.00	−0.02 to 0.81^c^	1.00	0.38	Almost perfect	Fair
Confidence level during the evaluation^a^	0.00–0.79^d^	0.09–0.62^c^	0.35	0.40	Fair	Fair

*Note:* Evaluation of standard‐ and low‐dose protocol cone‐beam computed tomography images by four observers for the parameters assessed at least 6 months, or more, following alveolar bone grafting. Abbreviations: LD = low‐dose protocol; SD = standard‐dose protocol.

^a^ Weighted kappa.

^b^ Intraclass correlation coefficient (ICC).

^c^ Kappas or ICCs are not significant in some of the observer combinations (*p* > 0.05).

^d^ Kappas cannot be calculated for some of the observer combinations due to data homogeneity.

Table 4 presents the kappas and ICCs for intra‐observer agreement. The agreement was almost perfect for all observers for bone integration and varied between good and excellent for graft height. For graft thickness, agreement ranged from substantial to almost perfect and was excellent for postoperative nasal floor height between sides. Intra‐observer agreement for subjective image quality and confidence level ranged from moderate to almost perfect with lower kappas for low‐dose protocol images.

**Table 4:** Intra‐observer agreement at a minimum interval of 1 month.

Assessed parameters	Observer							
	1		2		3		4	
	SD	LD	SD	LD	SD	LD	SD	LD
Graft integration^a^	0.95	0.95	1.00	1.00	0.95	0.96	1.00	1.00
Graft height^b^	0.88	0.85	0.95	0.98	0.97	0.96	0.99	0.97
Graft thickness—middle^a^	–c	1.00	–c	0.73	1.00	0.65	1.00	0.77
Graft thickness—periapical^a^	–c	1.00	1.00	1.00	1.00	1.00	0.61	1.00
Post‐operative nasal floor height difference between sides^b^	0.97	0.99	0.99	1.00	0.99	0.99	1.00	1.00
Subjective image quality^a^	–d	0.58	1.00	0.76	–d	0.43	1.00	0.84
Confidence level during the evaluation^a^	–d	–c	0.68	0.62	1.00	0.58	–d	0.73

*Note:* Evaluation of standard‐ and low‐dose protocol cone‐beam computed tomography images by four observers for the parameters assessed at least 6 months, or more, following alveolar bone grafting. Abbreviations: LD = low‐dose protocol; SD = standard‐dose protocol.

^a^ Weighted kappa.

^b^ Intraclass correlation coefficient.

^c^ Kappas are not significant (*p* > 0.05).

^d^ Kappas cannot be calculated due to data homogeneity.

## 4 Discussion

For most parameters of interest, the low‐dose protocol used in this study possessed adequate image quality for the postoperative CBCT healing assessment of ABG, thus supporting our hypothesis. Low‐dose protocols have enjoyed increasing popularity in recent years, and various diagnostic and treatment needs have been shown to benefit from their utility (Iskanderani et al. 2020; Cederhag et al. 2023; Lemberger et al. 2024; Ihlis et al. 2022). In particular, the management of pediatric patients requires the utmost care in optimizing radiographic examinations (Pauwels, Silkosessak, et al. 2014). Various methods for optimizing radiation doses include adjusting exposure settings (Charuakkra et al. 2023) and the size of the FOV as well as choosing a partial rotation mode, as previously demonstrated by Pauwels, Zhang, et al. (2014).

In the present study, tube current in the low‐dose protocol was reduced from 5 to 2 mA while maintaining a tube voltage of 90 kV. Pauwels et al. consider tube current (mA) reductions to be preferable to reducing the tube voltage (kV) (Pauwels, Silkosessak, et al. 2014). Tube current is a measure of the quantity of photons, and reducing it increases noise (Baumann et al. 2022). Tube voltage affects both the quantity and mean energy of photons, (Pauwels, Silkosessak, et al. 2014) and reducing tube voltage entails not only more noise than a reduction in tube current would but also a higher absorbed dose to the patient due to the lower energy of the photons (Pauwels et al. 2017). Thus, dose reduction should be prioritized, even at the expense of increased image noise (Pauwels et al. 2017).

A recent study reported satisfactory image quality for evaluating CBCT images taken both pre‐ and post‐ABG with a low‐dose protocol (Lemberger et al. 2024). However, in that study, patients underwent only one CBCT examination, either with a standard‐ or a low‐dose protocol. Thus, no direct comparison between low‐ and standard‐dose protocols was made for each patient. In contrast, the present study required patients to undergo the same CBCT examination twice, which is ethically challenging. However, as the standard‐dose protocol used in the present study had a tube current of 5 mA, and the low‐dose protocol had a tube current of 2 mA, the total dose received by the participants was approximately equivalent or less in comparison to that in a default protocol recommended by the manufacturer. The ICRP in Biomedical Research has published guidelines (International Commission on Radiological Protect 1992) for research with ionizing radiation on humans, which state that research is essential for exploring potential societal gains (International Commission on Radiological Protect 1992). The hypothesized potential future benefits of a low‐dose protocol—specifically, the potential reduction in cumulative radiation dose to this group of young people who are already more frequently exposed to ionizing radiation—are most likely well founded, as the ICRP guidelines presumed. Therefore, the potential benefits at the population level outweigh the potential risks to the individual (Cederhag et al. 2023).

The image quality of the low‐dose protocol images in our study was adequate for assessing all objective parameters necessary for evaluating the success of ABG (Wiedel et al. 2021; Kamperos et al. 2020; Suomalainen et al. 2014; Anver et al. 2020). However, in the subjective parameters, significant differences in perceived image quality (one observer) and confidence level in performing the evaluations (three observers) occurred. This variance may be attributed to the differences in perceived versus actual image quality required to attain consistent results when examining radiographic images among the various observers.

Assessment of graft integration and height, and differences between sides in postoperative nasal floor height, had high inter‐ and intra‐observer agreement. These are indications that the low‐dose protocol provides adequate image quality for analyzing these parameters.

However, graft thickness assessments had neither inter‐ nor intra‐reliabilities that were as strong. One explanation may be variations in the selection of CBCT slices for analyzing this parameter, which result in different graft thickness measurements across slices. It is important to point out that in clinical situations, oral and maxillofacial radiologists analyze the entire stack of images across the three planes to determine the thickness of the graft, rather than just at two points.

For subjective image quality, inter‐observer agreement was lower for the low‐dose protocol compared to the standard‐dose protocol. One explanation might be the higher noise level in the low‐dose images, which made it more difficult for some observers and lowered agreement. Confidence level had less than high inter‐observer agreement for both protocols.

Assessment of CBCT examinations is highly individual and relies on the experience of the observer in interpreting noisy images. Higher inter‐observer agreement occurred between the two oral and maxillofacial radiologists who had more experience with low‐dose protocols, as they were more comfortable analyzing images with a higher noise level. This is in line with the Lemberger et al. (2024) study that observed variations in how observers interpreted CBCT images in patients with orofacial clefts. They attributed the variations to personal preferences and varying levels of experience (Lemberger et al. 2024).

Lower kappas for intra‐observer agreement in subjective image quality and confidence level for the low‐dose protocol indicate that images with higher noise levels are more challenging to analyze, and observers may find it more difficult to make diagnostic decisions. The lower intra‐ and inter‐observer agreements on subjective image quality are consistent with findings in Charuakkra et al. (2023) and Ihlis et al. (2022). In fact, a CBCT image with a high noise level can elicit varying perceptions of visual appeal among different observers, as it is a subjective preference (Charuakkra et al. 2023).

As observed in the Iskanderani et al. study, dose reduction leads to higher noise levels, which degrade image quality and affect spatial and contrast resolution (Iskanderani et al. 2020). However, this does not imply that the observer will be unable to discern object details or make diagnoses (Iskanderani et al. 2020) as the present study demonstrates. It is worth noting that not all information available in the CBCT images of patients with orofacial clefts is clinically significant for diagnosis and treatment planning (Lemberger et al. 2024).

The present study assessed postgraft healing in a CBCT examination according to the study design. However, although CBCT is an excellent radiographic method for post‐graft controls, a 2D intraoral radiograph should be considered first; if graft failure or an open residual cleft is detectable clinically, or on a 2D intraoral radiograph, a CBCT examination would be unnecessary and should not be done (Wiedel et al. 2021). However, if healing is observed on 2D images, verification with a low‐dose protocol CBCT examination should be considered.

When a detailed analysis of anatomical structures is necessary, the CBCT protocol should strive for high‐resolution using higher exposure settings (Charuakkra et al. 2023). For larger anatomical structures, such as in orofacial cleft cases, a low‐dose CBCT protocol is suitable for treatment planning, outcome, and measurement accuracy (Oenning et al. 2018; Charuakkra et al. 2023). In fact, not all clinical situations require high image quality in the form of high‐resolution and noise‐free images (Iskanderani et al. 2020), which seems to be the case in post‐graft CBCT examinations.

Metal and motion artifacts were present in some of the CBCT examinations analyzed in the present study, which posed challenges for certain evaluations. The metal artifacts were primarily due to fixed orthodontic appliances, as Suomalianen et al. also observed (Suomalainen et al. 2014). Metal artifacts can be problematic in studies with radiographic or tomographic images (Garib et al. 2017). De Mulder et al. have documented motion artifacts, similar to those observed in the present study, in CBCT images of patients with orofacial clefts (De Mulder et al. 2018). However, this is reflective of typical conditions encountered in clinical practice.

Further efforts to optimize low‐dose CBCT protocols for other purposes within the oral and maxillofacial area have to be made to reduce the radiation doses provided to patients, according to European guidelines and regulations (European Commission 2012; Iskanderani et al. 2020; Cederhag et al. 2023; Lemberger et al. 2024; Ihlis et al. 2022).

### 4.1 Study Strengths

The present study was a clinical study, with real patients. In contrast, most studies investigating low‐dose protocols are performed on phantoms, cadavers, and dry skulls, which may not always offer clinically relevant evidence (Yeung, Jacobs, and Bornstein 2019). In particular, the assessment of subjective image quality can differ between studies conducted on phantoms and clinical studies (Charuakkra et al. 2023).

### 4.2 Study Limitations

The study cohort was small. Recruitment to our study from the pool of pediatric patients with orofacial clefts undergoing ABG at the Skåne University Hospital in Malmö, Sweden, was challenging due to the small numbers of these patients being treated annually. Consequently, the present results should be interpreted with caution. Additionally, the low‐dose protocol tested in this study was tailored for our clinic CBCT unit, which may pose challenges in the application of the protocol to other brands and models. In comparison to medical CT, it is not currently possible to quantify bone quality in CBCT using Hounsfield values due to the way the images are produced (Pauwels et al. 2015).

## 5 Conclusion

The low‐dose protocol yielded adequate image quality for postoperative CBCT healing assessment in patients who have undergone ABG. However, the confidence level of observers during the assessment with the low‐dose protocol was reduced.

## Author Contributions

**António Vicente:** study conception and design, material preparation, dataset collection, data analysis. **Josefine Cederhag:** study conception and design, data analysis. **Nilofar Rashidi:** study conception and design. **Anna‐Paulina Wiedel:** study conception and design. **Magnus Becker:** study conception and design. **Susanne Brogårdh‐Roth:** study conception and design, material preparation, dataset collection. **Xie‐Qi Shi:** study conception and design, material preparation, dataset collection, data analysis. **Kristina Hellén‐Halme:** study conception and design, material preparation, dataset collection, data analysis. All authors contributed to the writing of the manuscript.

## Ethics Statement

The Swedish Ethical Review Authority approved the present study (daybook no. [Dnr.] 2020‐06790), which followed the principles of the Declaration of Helsinki throughout. Our study design also followed the International Commission on Radiological Protection (ICRP) guidelines (International Commission on Radiological Protect 1992) for research projects. All participants and their guardians signed an informed consent form after receiving information about the protocol and the benefits of the study. The study is registered on ClinicalTrials.gov (NCT06395077).

## Conflicts of Interest

The authors declare no conflicts of interest.

## Data Availability

The datasets used and analyzed in the present study are available from the corresponding author upon reasonable request.

## References

[cre270021-bib-0001] Abramson, Z. R. , Z. S. Peacock , H. L. Cohen , and A. F. Choudhri . 2015. “Radiology of Cleft Lip and Palate: Imaging for the Prenatal Period and Throughout Life.” Radiographics 35, no. 7: 2053–2063. 10.1148/rg.2015150050. 26562237

[cre270021-bib-0002] Van Acker, J. W. G. , N. S. Pauwels , R. G. E. C. Cauwels , and S. Rajasekharan . 2020. “Outcomes of Different Radioprotective Precautions in Children Undergoing Dental Radiography: A Systematic Review.” European Archives of Paediatric Dentistry 21, no. 4: 463–508. 10.1007/s40368-020-00544-8. 32557182

[cre270021-bib-0003] Anver, T. D. , L. Mirzai , P. Li , K. K. Powell , and P. D. Waite . 2020. “Long‐Term Postoperative Cone‐Beam Computed Tomography Analysis of Secondary Bone Grafting in 79 Patients With Unrepaired Alveolar Clefts.” Journal of Oral and Maxillofacial Surgery 78, no. 7: 1164–1170. 10.1016/j.joms.2019.10.010. 31751522

[cre270021-bib-0004] Baumann, E. , M. M. Bornstein , M. Dalstra , C. Verna , and D. C. Dagassan‐Berndt . 2022. “Image Quality Assessment of Three Cone Beam Computed Tomography Scanners—An Analysis of the Visibility of Anatomical Landmarks.” European Journal of Orthodontics 44, no. 5: 513–521. 10.1093/ejo/cjac004. 35366310

[cre270021-bib-0005] Cederhag, J. , D. Iskanderani , P. Alstergren , X. Q. Shi , and K. Hellén‐Halme . 2023. “Visibility of Anatomical Landmarks in the Region of the Mandibular Third Molar, a Comparison Between a Low‐Dose and Default Protocol of CBCT.” Acta Odontologica Scandinavica 81, no. 6: 449–455. 10.1080/00016357.2023.2170462. 36748228

[cre270021-bib-0006] Charuakkra, A. , P. Mahasantipiya , A. Lehtinen , J. Koivisto , and J. Järnstedt . 2023. “Comparison of Subjective Image Analysis and Effective Dose Between Low‐Dose Cone‐Beam Computed Tomography Machines.” Dentomaxillofacial Radiology 52, no. 2: 20220176. 10.1259/dmfr.20220176. 36168973 PMC9974239

[cre270021-bib-0007] European Commission . 2012. *Radiation Protection N 172 Cone Beam CT for Dental and Maxillofacial Radiology. Evidence Based Guidelines*. Directorate‐General for Energy, Directorate D—Nuclear Energy, Unit D4—Radiation Protection. http://www.sedentexct.eu/files/radiation_protection_172.pdf.

[cre270021-bib-0008] Garib, D. , C. Massaro , M. Yatabe , G. Janson , and J. R. P. Lauris . 2017. “Mesial and Distal Alveolar Bone Morphology in Maxillary Canines Moved Into the Grafted Alveolar Cleft: Computed Tomography Evaluation.” American Journal of Orthodontics and Dentofacial Orthopedics 151, no. 5: 869–877. 10.1016/j.ajodo.2016.11.019. 28457264

[cre270021-bib-0009] Hung, K. F. , L. Hui , A. W. K. Yeung , R. Jacobs , Y. Y. Leung , and M. M. Bornstein . 2022. “An Analysis of Patient Dose Received During Cone‐Beam Computed Tomography in Relation to Scan Settings and Imaging Indications as Seen in a Dental Institution in Order to Establish Institutional Diagnostic Reference Levels.” Dentomaxillofacial Radiology 51, no. 5: 20200529. 10.1259/dmfr.20200529. 35230883 PMC10043612

[cre270021-bib-0010] Ihlis, R. L. , N. Kadesjö , G. Tsilingaridis , D. Benchimol , and X. Q. Shi . 2022. “Image Quality Assessment of Low‐Dose Protocols in Cone Beam Computed Tomography of the Anterior Maxilla.” Oral Surgery, Oral Medicine, Oral Pathology and Oral Radiology 133, no. 4: 483–491. 10.1016/j.oooo.2021.10.001. 34742681

[cre270021-bib-0011] International Commission on Radiological Protection (ICRP) . 1992. *Radiological Protection in Biomedical Research*. ICRP Publication 62. https://www.icrp.org/publication.asp?id=ICRP%20Publication%2062.

[cre270021-bib-0012] Iskanderani, D. , M. Nilsson , P. Alstergren , X. Q. Shi , and K. Hellen‐Halme . 2020. “Evaluation of a Low‐Dose Protocol for Cone Beam Computed Tomography of the Temporomandibular Joint.” Dentomaxillofacial Radiology 49, no. 6: 20190495. 10.1259/dmfr.20190495. 32250642 PMC7461740

[cre270021-bib-0013] Jacobs, R. , R. Pauwels , W. C. Scarfe , et al. 2018. “Pediatric Cleft Palate Patients Show a 3‐ to 5‐Fold Increase in Cumulative Radiation Exposure From Dental Radiology Compared With an Age‐ and Gender‐Matched Population: A Retrospective Cohort Study.” Clinical Oral Investigations 22, no. 4: 1783–1793. 10.1007/s00784-017-2274-0. 29188451

[cre270021-bib-0014] Kamperos, G. , N. Theologie‐Lygidakis , K. Tsiklakis , and I. Iatrou . 2020. “A Novel Success Scale for Evaluating Alveolar Cleft Repair Using Cone‐Beam Computed Tomography.” Journal of Cranio‐Maxillofacial Surgery 48, no. 4: 391–398. 10.1016/j.jcms.2020.02.003. 32127303

[cre270021-bib-0015] Ko, J. , S. Rustia , L. Alkharafi , R. Ganguly , S. L. K. Yen , and S. Oberoi . 2024. “Comparison of Alveolar Bone Grafting Outcomes Using CBCT in Individuals With UCLP Based on the Presurgical Orthodontic Treatment Methods.” Cleft Palate Craniofacial Journal 61, no. 5: 791–800. 10.1177/10556656221143945. PMC10981178 36748327

[cre270021-bib-0016] Koo, T. K. , and M. Y. Li . 2016. “A Guideline of Selecting and Reporting Intraclass Correlation Coefficients for Reliability Research.” Journal of Chiropractic Medicine 15, no. 2: 155–163. 10.1016/j.jcm.2016.02.012. 27330520 PMC4913118

[cre270021-bib-0017] Landis, J. R. , and G. G. Koch . 1977. “The Measurement of Observer Agreement for Categorical Data.” Biometrics 33, no. 1: 159–174. https://www.ncbi.nlm.nih.gov/pubmed/843571. 843571

[cre270021-bib-0018] Lemberger, M. , T. Regnstrand , A. Karsten , D. Benchimol , and X. Q. Shi . 2024. “Low‐Dose Cone‐Beam Computed Tomography for Assessment of Alveolar Clefts: A Randomized Controlled Trial in Image Quality.” Plastic and Reconstructive Surgery 153, no. 4: 897–903. 10.1097/PRS.0000000000010588. 37092973

[cre270021-bib-0019] De Mulder, D. , M. Cadenas de llano‐Pérula , R. Jacobs , A. Verdonck , and G. Willems . 2019. “Three‐Dimensional Radiological Evaluation of Secondary Alveolar Bone Grafting in Cleft Lip and Palate Patients: A Systematic Review.” Dentomaxillofacial Radiology 48, no. 1: 20180047. 10.1259/dmfr.20180047. 29947253 PMC6398910

[cre270021-bib-0020] De Mulder, D. , M. Cadenas de Llano‐Pérula , G. Willems , R. Jacobs , J. T. Dormaar , and A. Verdonck . 2018. “An Optimized Imaging Protocol for Orofacial Cleft Patients.” Clinical and Experimental Dental Research 4, no. 5: 152–157. 10.1002/cre2.123. 30386636 PMC6203823

[cre270021-bib-0021] Oberoi, S. , R. Chigurupati , P. Gill , W. Y. Hoffman , and K. Vargervik . 2009. “Volumetric Assessment of Secondary Alveolar Bone Grafting Using Cone Beam Computed Tomography.” Cleft Palate‐Craniofacial Journal 46, no. 5: 503–511. 10.1597/08-153.1. 19929098

[cre270021-bib-0022] Oenning, A. C. , R. Jacobs , R. Pauwels , et al. 2018. “Cone‐Beam CT in Paediatric Dentistry: Dimitra Project Position Statement.” Pediatric Radiology 48, no. 3: 308–316. 10.1007/s00247-017-4012-9. 29143199

[cre270021-bib-0023] Pauwels, R. , R. Jacobs , R. Bogaerts , H. Bosmans , and S. Panmekiate . 2017. “Determination of Size‐Specific Exposure Settings in Dental Cone‐Beam CT.” European Radiology 27, no. 1: 279–285. 10.1007/s00330-016-4353-z. 27108296

[cre270021-bib-0024] Pauwels, R. , R. Jacobs , S. R. Singer , and M. Mupparapu . 2015. “Cbct‐Based Bone Quality Assessment: Are Hounsfield Units Applicable?” Dentomaxillofacial Radiology 44, no. 1: 20140238. 10.1259/dmfr.20140238. 25315442 PMC4277442

[cre270021-bib-0025] Pauwels, R. , O. Silkosessak , R. Jacobs , R. Bogaerts , H. Bosmans , and S. Panmekiate . 2014. “A Pragmatic Approach to Determine the Optimal KVP in Cone Beam CT: Balancing Contrast‐to‐Noise Ratio and Radiation Dose.” Dentomaxillofacial Radiology 43, no. 5: 20140059. 10.1259/dmfr.20140059. 24708447 PMC4082271

[cre270021-bib-0026] Pauwels, R. , G. Zhang , C. Theodorakou , et al. 2014. “Effective Radiation Dose and Eye Lens Dose in Dental Cone Beam CT: Effect of Field of View and Angle of Rotation.” Br J Radiol 87, no. 1042: 20130654. 10.1259/bjr.20130654. 25189417 PMC4170857

[cre270021-bib-0027] Salari, N. , N. Darvishi , M. Heydari , S. Bokaee , F. Darvishi , and M. Mohammadi . 2022. “Global Prevalence of Cleft Palate, Cleft Lip and Cleft Palate and Lip: A Comprehensive Systematic Review and Meta‐Analysis.” Journal of Stomatology, Oral and Maxillofacial Surgery 123, no. 2: 110–120. 10.1016/j.jormas.2021.05.008. 34033944

[cre270021-bib-0028] Shkoukani, M. A. , M. Chen , and A. Vong . 2013. “Cleft Lip—A Comprehensive Review.” Frontiers in Pediatrics 1: 53. 10.3389/fped.2013.00053. 24400297 PMC3873527

[cre270021-bib-0029] Suomalainen, A. , T. Aberg , J. Rautio , and K. Hurmerinta . 2014. “Cone Beam Computed Tomography in the Assessment of Alveolar Bone Grafting in Children With Unilateral Cleft Lip and Palate.” European Journal of Orthodontics 36, no. 5: 603–611. 10.1093/ejo/cjt105. 24509615

[cre270021-bib-0030] Wiedel, A.‐P. , H. Svensson , K. Hellén‐Halme , H. Ghaffari , and M. Becker . 2021. “Two‐Dimensional Intra‐Oral Radiographs Compared to Three‐Dimensional Cbct at Six‐Month Post‐Operative Evaluation of Secondary Bone‐Grafting in Patients With Cleft Lip and Palate.” Journal of Dental Applications 7, no. 1: 451–455. 10.26420/jdentapp.

[cre270021-bib-0031] Yeung, A. W. K. , R. Jacobs , and M. M. Bornstein . 2019. “Novel Low‐Dose Protocols Using Cone Beam Computed Tomography in Dental Medicine: A Review Focusing on Indications, Limitations, and Future Possibilities.” Clinical Oral Investigations 23, no. 6: 2573–2581. 10.1007/s00784-019-02907-y. 31025192

